# Brain-derived extracellular vesicles potentially mediate crosstalk with peripheral organs in neurodegenerative diseases

**DOI:** 10.3389/fcell.2025.1710150

**Published:** 2026-01-06

**Authors:** Ramzi H. Hamdalla, Vibha B. Bhaskar, Changhai Tian

**Affiliations:** Department of Toxicology and Cancer Biology, University of Kentucky College of Medicine, Lexington, KY, United States

**Keywords:** brain, extracellular vesicles, inter-organ communication, neurodegenerative disorders, peripheral organs

## Abstract

Brain-Derived Extracellular vesicles (BDEVs) are emerging mediators of intra- and interorgan communication in neurodegenerative diseases (NDs) such as Alzheimer’s Disease (AD) and Parkinson’s Disease (PD). A growing body of evidence suggests that BDEVs play an important role in modulating intercellular communication within the central nervous system in the pathogenesis of many NDs. By transporting non-coding RNAs (e.g., miRNAs) and important pathological proteins, BDEVs also influence peripheral organs and contribute to the progression of disease in the central nervous system (CNS). This review extends the understanding of NDs beyond solely brain dysfunction and gives a novel framework for the progression of these diseases, uniquely emphasizing the currently underexplored mechanisms by which BDEV-mediated communication exacerbates or potentially initiates peripheral dysfunction or complications. It maps and clarifies the specific and potential mechanisms by which CNS-originating EV activity proliferates systemic dysfunction, presenting new opportunities and areas for therapeutic and diagnostic treatments for NDs. These findings are contextualized across multiple NDs, including Amyotrophic Lateral Sclerosis (ALS), Huntington’s Disease (HD), and Multiple Sclerosis (MS), by incorporating data on dysregulated BDEV miRNAs and toxic proteins to map the pathway of BDEV-mediated disease spread.

## 1 Introduction

Neurodegenerative diseases (NDs), including Alzheimer’s disease (AD), Parkinson’s disease (PD), are characterized by progressive neuronal dysfunction and loss, resulting in severe cognitive, motor, and behavioral impairments. Collectively affecting approximately 8 million to 9 million Americans according to current projections, the underlying causes of these disorders remain multifaceted, functioning through complex mechanisms (Rajan et al., 2021; Willis et al., 2022).

Brain-derived extracellular vesicles (BDEVs) are a subset of extracellular vesicles (EVs) that have garnered significant attention due to their potential as diagnostic biomarkers and therapeutic targets, as well as their ability to cross the blood-brain barrier (BBB) and serve as mediators of neuroimmune and neuroendocrine communication. There is significant evidence supporting the roles of EV types, such as exosomes, microvesicles, and apoptotic bodies, in mediating both local and remote neuronal function (Cabrera-Pastor, 2024). BDEVs can encapsulate and transport disease-associated molecules, including misfolded proteins, pro-inflammatory cytokines, and RNA species, into the venous system. Cargo that circulates to peripheral organs can potentially contribute to the etiology of NDs, in addition to inducing or exacerbating complications in other parts of the body (Raghav et al., 2022).

In the context of NDs, the role of BDEVs has been implicated in the propagation of pathogenic proteins such as amyloid-beta (Aβ) and alpha-synuclein (α-syn), which are hallmarks of AD and PD, respectively (Su et al., 2021; Guo et al., 2020). However, the precise mechanisms by which BDEVs mediate communications between the brain and peripheral organs, and their contributions to disease progression remain obscure, necessitating further investigation. Emerging evidence highlights the critical role of BDEVs as a mediator of interorgan communication, particularly involving the bidirectional signaling between the central nervous system (CNS) and peripheral systems such as the cardiovascular, endocrine, and urinary systems (Li et al., 2023a; Makrygianni and Chrousos, 2023).

This article discusses the role of BDEVs in mediating communications from the brain to peripheral organs in neurodegenerative diseases. We discuss their biogenesis, molecular cargo, and transport targets, with a focus on their connection to the pathogenesis of neurodegenerative diseases, and their observed and potential effects on other body systems. Understanding these mechanisms could provide novel insights into the systemic nature of neurodegenerative diseases and pave the way for innovative diagnostic and therapeutic approaches.

## 2 Extracellular vesicles overview

**FIGURE 1:**
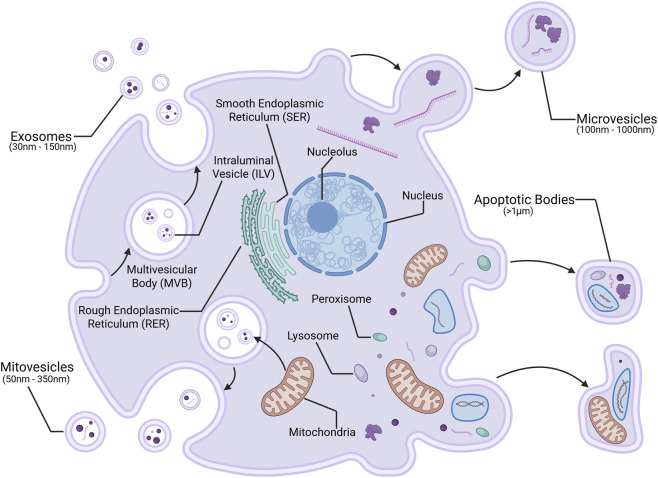
EV Biogenesis. Generated in Biorender.com. Illustration depicting the production of exosomes and microvesicles, which both may carry proteins, lipids, and nucleic acids; apoptotic bodies, carrying organelles and cellular debris; and mitovesicles, carrying proteins and nucleic acids, in a cellular membrane.

EVs are lipid bi-layer membrane-bound vesicles released by cells into the extracellular matrix. We review EVs that come in one of four forms as illustrated in Figure 1: exosomes, microvesicles, apoptotic bodies, and mitovesicles.

Exosomes are smaller (30 nm–150 nm) EVs that can contain an array of contents such as proteins, lipids, and nucleic acids including mRNAs and miRNAs. Exosome formation begins with the inward budding of an endosome from the lipid bi-layer membrane, which then matures into multivesicular bodies (MVBs) that carry intraluminal vesicles (ILVs). The MVB ultimately fuses with the cell membrane, releasing the ILVs as exosomes. Microvesicles (MVs) tend to be larger (100 nm–1000 nm) than exosomes, and like exosomes, typically carry proteins, lipids, and nucleic acids (Jeppesen et al., 2019). MV biogenesis is a result of the budding and pinching of the lipid bi-layer membrane, resulting in ectocytosis. Multiple common mechanisms and complex proteins are involved in the biogenesis of exosomes and MVs, including Rab GTPase, such as the ADP ribosylation factor 6 (ARF6) and Rab35 in MVs, Sytenin-1, tumor susceptibility gene 101 (TSG101), endosomal sorting complex required for transport (ESCRT), apoptosis-linked gene 2-interacting protein X (ALIX), soluble *N*-ethylmaleimide-sensitive factor (NSF) attachment protein receptor (SNARE), sphingomyelinases, tetraspanins, and ceramides (Kalluri and LeBleu, 2020; Clancy et al., 2021).

Apoptotic bodies, however, are created during apoptosis resulting in cell death. They are the largest type of EV (>1 μm) and tend to carry organelles and other cellular debris intended for phagocytosis (Jeppesen et al., 2023). Mitovesicles are a recently discovered type of EV (50 nm–350 nm), and while the specific mechanisms by which they are created are poorly understood, they are believed to form in a manner similar to exosomal MVBs, and carry mitochondrial components, including DNA, miRNA, and specific proteins. Studies have also reported their abundance in NDs, and their connection to mitochondrial dysfunction, suggesting that they may play a role in the propagation of neuroinflammation in NDs such as AD and PD (D'Acunzo et al., 2024).

In order to analyze and characterize EVs, they need to be isolated from the blood, cerebrospinal fluid, or other relevant bodily fluids. Brain tissue, or other tissues, can also be used as a source, allowing for EVs produced or found locally in specific organs to be isolated. EVs are most commonly isolated through ultracentrifugation (UC), however this process is time consuming and requires collecting large samples of EVs in order to isolate them. Alternate techniques are becoming more commonplace because of UCs shortcomings and limitations. Size-Exclusion Chromotagraphy (SEC) has been shown to yield higher purity EVs, and sucrose gradient UC has been presented as one of the highest purity options when analyzing brain tissues (Zhang et al., 2023b). Many recent studies advocate for utilizing multiple filtration and purification techniques in tandem in order to yield the highest purity EVs for study, as insufficient purification can lead to confounding results (Jank et al., 2025; Ovchinnikova et al., 2024). Once isolated, EVs are most commonly characterized through a series of Western blot or qRT-PCR tests, and the use of electron microscopy. Nanoparticle tracking analysis (NTA) tracks the motion of individual particles, allowing experimenters to quantify the yield and size distributions of EV samples (Collier et al., 2025). Modern high-sensitivity flow cytometry techniques allow for the robust results detailing particle concentration and size distributions, as well as phenotypic information on EV surface markers allowing for types of EVs to be differentiated (Jank et al., 2025; Kim et al., 2024a). Although not particularly routine, EVs can also be marked with antibodies, such as the Glast antibody which targets astrocyte-derived EVs, allowing for EV origin to be experimentally determined or confirmed (Figueroa-Hall et al., 2025). Additionally, multiple techniques can be utilized within the same procedure to collect a more complete characterization of collected EV samples, such as combining single-molecule flow cytometry and super-resolution microscopy to validate results (Jank et al., 2025; Ovchinnikova et al., 2024). EV isolation methods are constantly being restudied in order to come up with more efficient procedures, as this is a topic that sits at the core of almost any study into the role EVs play within the human body.

## 3 Extracellular vesicle-mediated crosstalk between brain and peripheral organs in neurodegenerative diseases

EVs play an important role in intercellular and interorgan communication. EVs can transfer bioactive molecules to target cells, thereby regulating and influencing various cellular functions. Gaining an understanding of their biogenesis and what contents they carry is critical for understanding their involvement in intercellular and interorgan communication and their impact on health and disease. Within the immune system, EVs have been proven to regulate immune responses. Immune cells, such as dendritic cells and macrophages, secrete EVs enriched with immunomodulatory molecules which can alter the function and behavior of nearby immune cells, coordinating immune responses (Marar et al., 2021).

In the nervous system, BDEVs play an important role in CNS processes, including synaptic plasticity, modulation of neuroinflammation, and neuronal survival (Pascual et al., 2020). They modulate intercellular signaling by transferring bioactive molecules such as microRNAs, proteins, and lipids capable of influencing neuronal development and connectivity. Dysfunctional BDEV signaling has been implicated in neurodegenerative diseases, where they may propagate toxic protein aggregates known to correlate with neurodegenerative diseases. BDEVs from ND patients have been found to carry altered miRNA profiles, as shown in Table 1, with both AD and PD BDEVs carrying toxins such as Amyloid-β (Aβ) and α-synuclein (α-syn), respectively, which feature heavily in their pathology (Vandendriessche et al., 2020). BDEVs can control the permeability and integrity of the BBB and can function as mediators between the brain and other organs due to their natural ability to cross the BBB, making them a hot topic in recent studies that we will be reviewing (Li et al., 2024b; Li et al., 2023b; Hosseinkhani et al., 2023).

**TABLE 1:** EV cargos and their potential contributions to brain and peripheral organ communication in AD and PD.

EV cargo	Variation	Affected organ	Functionalities in peripheral organ	Sample type	References
Aβ	↑ AD	Heart	Diminish ejection fractions, promote hypertrophy, increased risk of HF	Serum	Li et al. (2020a)
				CSF	Jia et al. (2021)
miR-483-5p	↓ AD	Bone/Adipose	Increase adipogenic differentiation	Serum	Tan et al. (2014)
				Plasma	Liu et al. (2024)
miR-133	↓ AD	Heart	Promote ventricular fibrillations, increased risk of MI	PFC	Han et al. (2024a)
				*In-Vitro* NPC	Marzano et al. (2019)
				AC, MO	Nelson et al. (2018)
miR-10a-5p	↑ AD	Heart	Inhibit hypertrophy, reduce build-up of atherosclerosis, decreased risk of HF	Whole Blood	Wu et al. (2020)
				CSF, PFC, Serum, HIP, FG, TG, Whole Blood	de Lourdes Signorini-Souza et al. (2024)
				CSF	Jia et al. (2021)
miR-342-3p	↓ AD	Heart, Kidney	Promote hypertrophy, increased risk of HF, increases kidney inflammation and fibrosis	Plasma, Serum, HIP	de Lourdes Signorini-Souza et al. (2024)
α-syn	↑ PD	Kidney, Gut, Liver	Prevents renal fibrosis, increases the risk of gastrointestinal dysfunction and liver disease	Plasma	Chan et al. (2023)
Aβ	↑ PD	Heart	Diminish ejection fractions, promote hypertrophy, increased risk of HF	Plasma	Chan et al. (2023), Wang et al. (2023)
miR-24	↑ PD	Bone/Adipose	Increases insulin resistance, increases adipogenic differentiation	Plasma	Kim et al. (2024b)
				Plasma, Serum	Barbagallo et al. (2020)
				CSF, Serum	Tong et al. (2022)
miR-29c	↑ PD	Bone/Adipose	Increases adipogenic differentiation	Plasma	Kim et al. (2024b)
				Serum	Ozdilek and Demircan (2021)
				CSF, Serum	Tong et al. (2022)
mi-124	↓ PD	Heart	Increased risk of HF	PFC	Ghafouri-Fard et al. (2021)
				*In vitro* SH-SY5Y, Plasma	Arora et al. (2025)
				Plasma	Angelopoulou et al. (2019)
miR-133b	↓ PD	Heart	Promote ventricular fibrillations, increased risk of MI	Plasma	Arora et al. (2025)
miR-195-3p	↑ PD	Heart	Increases cardiac fibrosis and hypertrophy, decreases cardiac contractility	Plasma	Kim et al. (2024b)
LRRK2	↑ PD	Kidney	Promotes renal epithelial cell apoptosis, exacerbating AKI	CSF	Karayel et al. (2022)

Abbreviations: EV, extracellular vesicle; AD, Alzheimer’s Disease; PD, Parkinson’s Disease; Aβ, Amyloid β; α-syn, α-synuclein; LRRK2, Leucine-rich repeat kinase 2; HF, heart failure; MI, myocardial infarction; AKI, acute kidney injury; CSF, cerebrospinal fluid; PFC, prefrontal cortex; NPC, neural progenitor cells; AC, anterior cingulate; MO, primary motor cortex; HIP, hippocampus; FG, frontal gyrus; TG, temporal gyrus; SH-SY5Y, SH-SY5Y, human neuroblastoma cell line.

The mechanism by which BDEVs impact the pathology of peripheral organs includes the direct transfer of cargo through receptor-mediated endocytosis, direct membrane fusion with certain target cells, and the indirect modulation of systemic inflammatory and immune responses. miRNAs that are transported via BDEVs typically regulate gene expression through silencing target mRNAs, whereas proteins like Aβ and α-synuclein (α-syn) directly contribute to aggregate formations and mitochondrial dysfunction in the peripheral tissues. Additionally, lipid cargos carried by these EVs may disrupt cellular membranes and signaling cascades further, leaving potential for organ dysfunction. Su et al. demonstrated that BDEVs from the human frontal cortex are enriched in plasmalogen phosphatidylethanolamine, sphingomyelin, and ether phosphatidylserine, which are lipids that are dysregulated in Alzheimer’s disease and suggest potential for peripheral lipid biomarker development (Su et al., 2021). Although this manuscript primarily focuses on miRNA and protein mediated mechanisms, we acknowledge the potential significance of non-miRNA or protein cargos, particularly lipids, which warrant further study.

### 3.1 Alzheimer’s disease

Alzheimer’s disease is a progressive neurodegenerative disorder, and the most common type of dementia. AD pathology is complex, affected by both genetic and environmental factors, and its symptoms include cognitive decline, memory impairment, and inability to perform daily tasks. The most common features of AD pathology include amyloid plaques, intracellular neurofibrillary tangles (NFTs), neuroinflammation, and loss of synaptic and neuronal ability (Orobets and Karamyshev, 2023). AD symptoms go beyond the CNS as well, correlating with complications in peripheral organs, including the heightened risk of heart failure and renal dysfunction, as well as mild risks for osteoporosis and other chronic conditions. Many recent studies have not only narrowed down the most useful and consistent biomarkers for identifying AD, but have also shed light on the sources of these biomarkers, and the mechanisms by which they move around and expedite the development of AD. EVs are largely influential in the progression of AD, being responsible for the transportation and circulation of Amyloid-β 42 (Aβ_42_) and the propagation of tau pathology (Vandendriessche et al., 2020).

Neuroinflammation is a common biomarker in neurodegenerative diseases, and is known to exacerbate neuronal injury and accelerate AD progression. Upon encountering Aβ aggregates, microglia transition to a pro-inflammatory state, releasing cytokines and triggering pathways for the phagocytosis of Aβ plaques. While this response initially aids in Aβ clearance, chronic activation can damage surrounding neurons, and can induce reactive astrocytes to contribute to Aβ-induced neuroinflammation (Hansen et al., 2018). Aβ aggregation precedes tau pathology and is thought to trigger tau hyperphosphorylation and aggregation, which contributes to the formation of NFTs, which in turn further drives neurodegeneration and cognitive decline (Ruan et al., 2021). Tau proteins act as stabilizers for the microtubules in axons, but, under abnormal physiological conditions, may experience hyperphosphorylation. This results in the aggregation of tau proteins and NFT formation, which have been shown to activate microglia and further compromise neuronal communication, triggering excessive neuroinflammatory responses that exacerbate neuronal damage. This neuroinflammation promotes further tau phosphorylation and aggregation, creating a vicious cycle of neurodegeneration (Chen and Yu, 2023).

**FIGURE 2:**
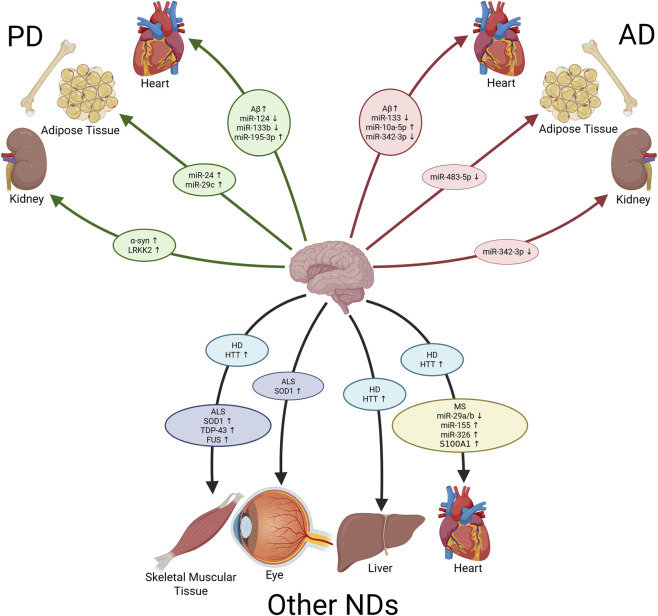
Potential BDEV Cargo Paths in NDs. Generated in Biorender.com. Schematic showing the proposed paths and destinations for various potentially brain-derived extracellular vesicle cargos to influence specific organs in various neurodegenerative disorders. AD, Alzheimer’s Disease; PD, Parkinson’s Disease; ND, Neurodegenerative Disease; Aβ, Amyloid β; α-syn, α-synuclein; LRRK2, Leucine-rich repeat kinase 2; SOD1, 762 Superoxide Dismutase 1; TDP-43, Transactive Response DNA-Binding Protein 43; FUS, Fused in Sarcoma; HTT, Huntingtin Protein.

Past studies have highlighted the role of EVs in influencing Aβ dynamics. The enzyme neutral sphingomyelinase 2 (nSMase2) facilitates the biogenesis of exosomes, playing a key role in the budding of ILVs into MVBs. *In vivo* AD mouse models that were treated with GW4869, an inhibitor of nSMase2 which thusly prevents EV secretion, showed reduced levels of Aβ_42_, and a reduction in the number of amyloid plaques in the brain, suggesting that EV secretion plays a significant role in the accumulation and aggregation of Aβ_42_ in AD pathology (Dinkins et al., 2014). Studies have shown that EVs gathered and isolated from the brain tissue of mice suffering from AD were significantly enriched in epitope-specific tau oligomers. These EVs were then proven to be capable of spreading misfolded tau *in vitro*, propagating tau pathology across neuronal networks, and accelerating the progression of AD (Ruan et al., 2021). Post-mortem samples of brain tissue taken from human AD patients have similarly shown that increased levels of EVs positively correlate with Aβ_42_ oligomers, suggesting that EVs are centrally involved in the progression of AD, and that BDEVs can transport biologically potent molecules to distant organ tissues (Song et al., 2020), such as the ones shown in Figure 2.

#### 3.1.1 Brain-derived EV-mediated crosstalk between the brain and skeletal and adipose tissue in Alzheimer’s disease

Recent studies reveal that AD BDEVs can promote shifts in bone marrow stem cells, regulating bone marrow stem cell (BMSC) differentiation from osteogenesis to adipogenesis and inducing bone marrow obesity. Liu et al. reported that AD BDEVs were enriched with miR-483-5p and worked independently of osteoclast activity to directly induce bone loss by affecting the fate of BMSCs, whereas bone loss was not present in control BDEV trials (Liu et al., 2023). It is evidenced that miR-483-5p promotes adipogenesis in BMSCs, with the expression of miR-483-5p positively correlating with bone loss and osteoporosis (Wang et al., 2022). miR-483-5p is known to suppress insulin-like growth factor 2 (IGF2), a gene critical for bone formation and the reduction of fat development in BMSCs (Li et al., 2020b). IGF2 inhibition leads to weaker bones with reduced mineralization, IGF2 overexpression can enhance bone metabolism and downregulate adipogenesis (Koshta et al., 2024). This shows that in an AD environment, BDEVs transported to distant organs can upregulate the differentiation of adipose tissue, emphasizing the important role of EVs as a mediator of interorgan communication across the brain-bone-adipose axis.

#### 3.1.2 Brain-derived EV-mediated crosstalk between the brain and heart tissue in Alzheimer’s disease

EV-mediated signaling is an important aspect of the brain-heart axis, and has significant implications for cardiovascular health and disease. BDEVs serve as carriers of pathological molecules, including Aβ and miRNAs, that can influence cardiac function, and accumulating evidence links Aβ pathology to cardiac abnormalities (Schaich et al., 2019). Troncone et al. reported increased concentrations of Aβ deposits in the heart tissue of AD patients, but not in control patients. Moreover, echocardiographic measurements also indicated that patients with AD had a higher risk of cardiac dysfunction (Troncone et al., 2016). While these findings suggest peripheral Aβ accumulation in AD patients, the specific contribution of BDEVs in delivering Aβ to heart requires further verification. More recently, Hall et al. found that Aβ_42_ reduces glucose clearance and impairs cardiac function, impacting mitochondrial metabolism pathways and inhibiting complex I (Hall et al., 2024). This effect was especially pronounced in obese mice. Similarly, Zhu et al. reported that elevated plasma Aβ levels are associated with diminished cardiac function, with increased Aβ levels correlating with lower left ventricular ejection fractions, increased left ventricular mass, and increased risk of heart failure in the general population (Zhu et al., 2023).

Repeated studies have shown that exosomal miR-133 expression within the brain is consistently downregulated in AD patients, and may protect neurons by preventing Aβ-induced cell apoptosis (Han et al., 2024a). Additionally, miR-133 is known to offer similar protective effects against myocardial infarction (MI). Investigations have consistently reported that miR-133 attenuates myocardial apoptosis and reduces infarction severity *in vivo* in mice. Olson et al. showed that miR-133 targets and suppresses caspase-induced apoptosis and oxidative stress, helping safeguard normal myocardial function. Its downregulation in patients with myocardial infarctions is correlated with cardiovascular complications and increased symptom severity (Olson et al., 2024). This evidence suggests an established EV-mediated interorgan communication link across the brain-heart axis, which may be negatively affected by the decrease in BDEV miR-133 levels in an AD environment, thereby increasing the risk of cardiovascular disease in patients. However, the origin of miR-133 found in blood samples has not been analyzed and needs further investigation.

Despite some contradictory evidence, EVs in the blood samples of AD patients have repeatedly reported downregulated levels of miR-342-3p, an miRNA expressed in endothelial, glial cells, and neuronal cells that has long since been theorized to be involved in inflammatory pathways and vascular dysfunction (de Lourdes Signorini-Souza et al., 2024; Giudici et al., 2025). Downregulated levels of miR-342-3p have been shown to correlate with endothelial dysfunction, as well as mediating the interactions with immune cells, influencing dendritic cell function and promoting cardiac inflammation, negatively affecting cardiovascular health as a result (Zhu et al., 2022; Mone et al., 2023). By studying the effect of miR-342-3p in mice, Chen et al. recognized that this miRNA regulates cardiac hypertrophy and cell growth (Chen et al., 2022). Further studies also indicated that dysregulated exosomal miR-342-3p levels contribute to heart failure pathophysiology. This is most likely a result of the impact of miR-342-3p’s reported role in cardiac repair, energy metabolism, and its involvement in inflammatory pathways and fibrosis (Wang et al., 2021; Vilella-Figuerola et al., 2022).

Studies into AD pathology have also reported increased levels of miR-10a-5p in EVs (Wu et al., 2020; de Lourdes Signorini-Souza et al., 2024; Jia et al., 2021), which is commonly considered to have a significant role in various cardiac pathologies, modulating processes such as cardiac fibrosis, myocardial cell survival, and inflammatory responses through multiple pathways. Cao et al. showed that miR-10a-5p protects heart cells during MI by inhibiting EGR2 and reducing apoptosis in cardiomyocytes. Furthermore, upregulated levels of miR-10a-5p have been shown to inhibit hypertrophy by downregulating T-box 5 (TBX5) (Cao et al., 2021). Alternatively, other studies have shown that miR-10a-5p is downregulated in atherosclerotic conditions, and that introducing EVs carrying miR-10a-5p cargo can help reverse these complications by normalizing conditions for endothelial progenitor cells (Alexandru et al., 2020). MI environments display decreased miR-10a-5p expression, whereas increasing its expression helped ameliorate cardiac conditions by mediating the miR-10a-5p/IGLON5/LMX1A pathway (Xiong et al., 2021). These findings suggest that in an AD environment, BDEV-transported miR-10a-5p may play a cardioprotective role. In AD, BDEVs may carry dysregulated levels of miRNAs and Aβ_42_, contributing to cardiovascular damage while offering limited cardioprotective benefits under certain conditions. Collectively, these studies indicate that Aβ, miR-342, miR-133, and miR-10a-5p may all function as messengers between the CNS and the heart. Although, while several of these studies report altered EV miRNA and protein levels in the peripheral blood of AD patients, many do not explicitly confirm CNS origin or track EV transit across the BBB, such as miR-133 which lacks strong evidence to firmly connect BDEV activity with the dysregulated EV levels seen in AD. Additionally, the connection between dysregulated BDEV levels and the cardiovascular complications that are known to be commonplace in AD pathology are primarily correlative and require further investigation to establish a causal relationship.

#### 3.1.3 Brain-derived EV-mediated crosstalk between the brain and kidney tissue in Alzheimer’s disease

BDEVs have been shown to play a role in mediating kidney health through miRNAs that drive these protective effects. The dysregulated miRNA profiles observed in AD suggest a potential pathway through which BDEVs may impact renal function. In addition to the role it plays in the heart, miR-342-3p, which we previously discussed as a downregulated protein in AD patients, also plays a role in the kidney (de Lourdes Signorini-Souza et al., 2024). Jiang et al. identified miR-342-3p as a mediator of fibrosis pathways, further emphasizing its protective role in renal pathology. The study demonstrated in mice that miR-342-3p downregulated SOX6, which plays a role in the promotion and transcription of key genes involved in fibrosis and kidney damage, such as TGF-β1, FN, C-IV, and PTEN (Jiang et al., 2020). Zheng et al. also found that miR-342-3p was heavily downregulated in rats with diabetic nephropathy, and revealed that miR-342-3p directly targets Caspase1, decreasing the expression of pro-inflammatory markers such as Caspase1, NLRP3, IL-18, and IL-1β, thus inhibiting pyroptosis and suppressing inflammation-driven cell death in renal tubular epithelial cells. Furthermore, introducing additional EVs carrying miR-342-3p was found to alleviate diabetic nephropathy symptoms (Zheng et al., 2023). Yang et al. provided more evidence that miR-342-3p inhibits IL-1β, IL-18, and TGF-β, thus inhibiting cell pyroptosis, and when miR-342-3p was downregulated, IL-1β and IL-18 levels were increased and the TGF-β/SMAD axis was activated, increasing inflammation and fibrosis (Yang et al., 2023b). The combined evidence suggests that BDEVs may play an important role in mediating kidney health and protecting against renal complications. The dysregulation of miRNA profiles in AD may exacerbate renal inflammation and fibrosis, contributing to the prevalence of chronic kidney disease in neurodegenerative diseases.

### 3.2 Parkinson’s disease

Parkinson’s disease (PD) is the most common progressive neurodegenerative disorder after Alzheimer’s disease. PD’s most common symptoms tend to relate to the degradation of motor control in patients, including symptoms such as tremors, bradykinesia, muscular rigidity, and postural instability. PD is characterized by the degeneration of dopamine-producing neurons in the substantia nigra, and the formation of aggregates of the synaptic protein α-synuclein (α-syn), termed lewy bodies (LBs) and lewy neurites (LNs), in surviving neurons (Morris et al., 2024). PD has been shown to correlate with mutations in genes such as SNCA, LRRK2, and PARK2, suggesting that these genes may play an influential role in the etiology of PD (Tolosa et al., 2021). Additional PD symptoms include strong links to cardiovascular, metabolic, and gastrointestinal diseases such as cardiac arrhythmias, diabetes, and constipation, and milder links to liver and kidney diseases, largely believed to be due to the disrupted autonomic nervous system function (Gonçalves et al., 2022).

α-synuclein is a protein abundantly found within the brain and is encoded by the SNCA gene. Under ordinary physiological conditions, α-syn takes a helical form and is responsible for the regulation of synaptic vesicle traffic and the release of neurotransmitters. The misfolding and aggregation of α-syn is a major part of PD pathology, resulting in the formation of neurotoxic LBs (Gao et al., 2023b). It is believed that α-syn misfolding propagates in a prion-like manner, spreading throughout the nervous system and causing widespread neurodegeneration, and recent studies have suggested that EVs hold an influential role in this process (Akbari-Gharalari et al., 2024).

#### 3.2.1 Brain-derived EV-mediated crosstalk between the brain and skeletal and adipose tissue in Parkinson’s disease

Recent findings suggest that BDEVs in PD may influence adipose and skeletal tissue, primarily through miRNAs including miR-24 and miR-29c, though the CNS origin of these EVs is not always experimentally confirmed. There is a clear correlation between miR-24 expression and metabolic dysfunction in human abdominal adipose tissue, with elevated levels of miR-24 observed in individuals suffering from obesity and both type 1 and type 2 diabetes (Mukherjee et al., 2022). It was suggested that miR-24 may contribute to insulin resistance due to its role in modulating inflammatory pathways and lipid metabolism. Adipose tissue remodeling in an obese macro environment is accompanied by macrophage infiltration and proliferation, which contribute to chronic inflammation and insulin resistance. Sprenkle et al. implicated that the miR-23-27-24 cluster plays a role in driving lipid-associated macrophage proliferation in obese adipose tissue. Specifically, it was reported that miR-24 enhances macrophage proliferation in mice by targeting the Eif4ebp2 gene, which mediates protein synthesis and proliferation in macrophages (Sprenkle et al., 2023).

Multiple studies have shown miR-24’s effect on the differentiation of mesenchymal stem cells (MSCs), and the role it plays in promoting adipogenesis and inhibiting osteogenesis. Jin et al. suggested that miR-24 downregulates the MAPK7 signaling pathway, a known inhibitor of adipogenesis, thus increasing the expression of pro-adipogenic markers, such as PPARγ, SREBP1, and ACC1 (Jin et al., 2016). Early findings in Zhao et al.’s investigation into the role miR-24 plays during the differentiation of MSCs reported that miR-24 was significantly upregulated during adipogenic differentiation. Further studies demonstrated that the overexpression of miR-24 in MSCs inhibited osteogenic differentiation, and that suppressing miR-24 reversed this trend, promoting osteogenic differentiation. Further analysis provides evidence that miR-24 directly targets T cell factor-1 (TCF-1), a transcription factor known to be involved in the regulation of bone development through its role in the Wnt signaling pathway (Zhao et al., 2015). Thus, miR-24 enhances the expression of adipogenic markers and facilitates the differentiation process by suppressing TCF-1 in bone MSCs. More recently, Zhang et al. elucidated the role of miR-29c in porcine MSCs, finding that miR-29c overexpression promoted adipogenic proliferation and differentiation, whereas its inhibition had the opposite effect, suppressing these processes (Zhang et al., 2024).

Studies report that BDEVs can carry miR-24 and miR-29c (Akers et al., 2015; Li et al., 2022), and may influence distant tissues by crossing the BBB. EVs isolated from the blood samples of PD patients have repeatedly shown upregulated levels of these miRNAs (Kim et al., 2024b; Barbagallo et al., 2020; Ozdilek and Demircan, 2021; Tong et al., 2022). However, evidence directly demonstrating BDEV-mediated delivery of these miRNAs to adipose or bone tissue is currently lacking. Nevertheless, these findings point to the possible role of BDEVs in promoting systemic insulin resistance, adipose tissue remodeling, and bone marrow adiposity, which are factors frequently observed in PD patients. This further elucidates the important role of EVs as mediators of interorgan communication across the brain-bone-adipose axis and their potential to cause complications in a PD macroenvironment.

#### 3.2.2 Brain-derived EV-mediated crosstalk between the brain and heart tissue in Parkinson’s disease

Similarly to AD pathology, levels of miR-133b are downregulated in PD BDEVs. This is linked to dopamine dysregulation and increased oxidative stress, factors that are critical to patient outcomes in cardiovascular diseases (Arora et al., 2025). Recent studies indicated that dysregulated miR-133b results in increased cardiovascular risk. By targeting pro-apoptotic and hypertrophic pathways, miR-133b preserves cardiac structure and function under stressful conditions, providing beneficial cardioprotective effects (Cibi et al., 2020; Crocco et al., 2024). As previously discussed in part 3.1.2, miR-133 confers cardioprotective effects by targeting and suppressing caspase-induced apoptosis, combating oxidative stress, and attenuating MI pathology. Furthermore, decreased levels of miR-133b in MI patients correlate with ventricular fibrillations and poorer outcomes in cardiovascular diseases (Olson et al., 2024; Crocco et al., 2024).

Under ordinary conditions, miR-124 protects cardiomyocytes from doxorubicin-induced cardiac injury by inhibiting p66Shc, a critical mediator of oxidative stress, thus reducing ROS production (Liu et al., 2020). Conversely, elevated circulating miR-124 has been implicated in exacerbating heart failure (HF) due to its anti-angiogenic effects. Zhao et al. showed that miR-124 inhibits tetraspanin CD151, a protein that facilitates angiogenesis, impairing vascularization in failing hearts (Zhao et al., 2018). Further studies have shown that increased levels of miR-124 correlate with poorer patient outcomes in acute stroke patients (He et al., 2019). Although further exploration is necessary, current evidence suggests that miR-124 exacerbates cardiac disease and its downregulation during PD may be protective (Angelopoulou et al., 2019; Ghafouri-Fard et al., 2021).

Most recently, biomarker studies showed elevated expression of miR-195-3p in PD EVs isolated from the blood (Kim et al., 2024b), an miRNA commonly expressed in neurons which has also been identified as a pivotal player in post-infarction cardiac remodeling and hypoxia-induced injury (He et al., 2021). According to Carvalho et al. elevated miR-195-3p levels exacerbate cardiac fibrosis and compromise heart function post-MI, while silencing miR-195-3p expression in animal models conversely resulted in reduced fibrosis and improved left ventricular function post-MI. Mechanistically, miR-195-3p targets genes regulating fibrosis, cell survival, hypertrophy, contractility, and metabolism (e.g., PTEN/AKT), underscoring its role in exacerbating cardiac damage (Carvalho et al., 2023). Furthermore, Isoflurane-mediated inhibition of miR-195-3p was similarly found to attenuate apoptosis and oxidative stress in cardiomyocytes, enhancing the survival of cardiomyocytes under hypoxic stress (Han et al., 2024b). Zhang et al. found that miR-195-3p directly downregulates Brain-Derived Neurotrophic Factor (BDNF), leading to impaired activation of the P-ERK1/2 signaling pathway, which is essential for cellular repair mechanisms (Zhang et al., 2022). It has been shown that upregulation of BDNF counteracts the detrimental effects of chronic ischemic injury, and thus elevating BDNF levels by inhibiting miR-195-3p was shown to enhance cardiomyocyte survival, improve cardiac contractility, and reduce pathological remodeling (Cannavo et al., 2023).

Collectively, these studies suggest that dysregulated BDEV miRNAs in PD significantly impact cardiovascular disease outcomes by promoting cardiac injury and complications. However, as with previous studies, further investigation is needed to experimentally confirm the CNS origin of these EVs, and to directly link their causality with their correlated symptoms.

#### 3.2.3 Brain-derived EV-mediated crosstalk between the brain and other peripheral organs in Parkinson’s disease

Studies have recorded increased exosomal levels of both LRRK2 and α-syn in PD cases, with some suggesting a potential brain-to-kidney EV-mediated signaling axis (Karayel et al., 2022; Chan et al., 2023). While the evidence is still emerging, it indicates the possibility of LRRK2 and α-syn enriched EVs traveling from the CNS to peripheral organs. LRRK2, a kinase involved in mitochondrial homeostasis, and has been shown to exacerbate acute kidney injury (AKI), by degrading Mitofusin-2 (MFN2) leading to mitochondrial fragmentation and impaired bioenergetics. This degradation disrupts mitochondrial integrity, promoting renal tubular epithelial cell apoptosis and contributing to AKI (Zhang et al., 2023a). However, it still remains unclear whether these effects on the periphery result from locally expressed or brain-derived EV-associated LRRK2, warranting further investigation. Bozic et al. suggests that α-syn regulates cellular stress responses and inflammation, reducing fibrotic signaling pathways. Knockdown of α-syn in renal tubular cells exacerbated fibrosis, while overexpression had a protective effect, implying that α-syn is a crucial modulator of kidney fibrosis (Bozic et al., 2020). Conversely, Braun et al. highlighted that α-syn accumulation in podocytes contributes to cellular injury and dysfunction. The pathological buildup of α-syn was linked to lysosomal impairment, increased oxidative stress, and apoptosis in podocytes, contrasting its potential protective role in renal tubular cells (Braun et al., 2023). Although both findings point to α-syn’s influence on renal function impairment, the mechanism of α-syn delivery, particularly if it is transported by BDEVs, is yet to be explicitly stated and needs to be clarified.

Multiple studies have also explored the effects of α-syn across the Brain-Gut and Brain-Liver Axes. McFleder et al. proposes that CD11c (+) antigen-presenting cells facilitate α-syn transport from the brain to the intestines, potentially contributing to gastrointestinal dysfunction commonly observed in PD patients. The recorded presence of α-syn in enteric neurons and its association with inflammation further underscore its potential role in gastrointestinal symptoms such as constipation, which often precede the hallmark motor symptoms of PD (McFleder et al., 2023). Reyes et al. highlights the liver’s involvement in processing and detoxifying α-syn, and claims that hepatic clearance mechanisms may mitigate neurodegeneration by reducing the impact of α-syn on the CNS by degrading α-syn aggregates, while suggesting that excessive α-syn accumulation in the liver may contribute to dysfunction and pathology (Reyes et al., 2021). This may imply that there is a liver-protective buffering role, though there is a lack of direct evidence of EV-mediated α-syn delivery to hepatocytes. Liver diseases such as nonalcoholic steatohepatitis (NASH) have also been linked to α-syn pathology. Kakimoto et al. reported a strong correlation between increased levels of α-syn and NASH and provided evidence that α-syn contributes to hepatic inflammation and fibrosis, suggesting a potential interaction between metabolic disorders and neurodegeneration mediated by the presence of α-syn in hepatocytes (Kakimoto et al., 2023). Post-translational modifications of α-syn, such as phosphorylation, nitration, and ubiquitination, are critical in modulating its function and aggregation propensity. Hallbeck et al. found that in PD mice, post-translational modifications in hepatic α-syn mirrored that of α-syn from the brain, which was believed to be a result of EV-mediated α-syn transportation across the Brain-Liver axis, suggesting a direct mechanistic link between neurodegenerative disease progression and liver dysfunction (Hallbeck et al., 2024). Yang et al. demonstrated that α-syn may also play a protective role as a potential anti-tumorigenic in hepatocellular carcinoma by interacting with metabotropic glutamate receptor 5 (mGluR5) and γ-synuclein, leading to the inhibition of tumorigenesis (Yang et al., 2023a). This suggests that α-syn’s function and effects may vary depending on the cellular context and disease state in various peripheral organs. Together, these findings highlight the complex roles of α-syn and LRRK2 in peripheral organ system functioning, and possibly point to the increased levels of these cargos in BDEVs as a unifying mechanism in the propagation of PD. Further studies are needed to confirm direct EV-mediated transport routes, identify specific cargo signatures, and clarify how the peripheral EV uptake influences subsequent disease progression.

### 3.3 Other neurodegenerative diseases

In addition to AD and PD, other NDs also dysregulate BDEV cargo but lack the prerequisite foundations required to elucidate the full possible extent of symptoms influenced by BDEV-mediated interorgan communication (See Table 2).

**TABLE 2:** EV cargos and their potential contributions to brain and peripheral organ communication in other NDs.

EV cargo	Variation	Affected organ	Functionalities in peripheral organ	Sample type	References
miR-29a/b	↓ MS	Heart	Induces hypertrophy, and increases the risk of atrial fibrillation and HF	Whole Blood	Yousuf and Qurashi (2021)
miR-155	↑ MS	Heart	Increases risk of CAD	Whole Blood	(Yousuf and Qurashi, 2021; Basak et al., 2023)
				Serum	Abaas and Darweesh (2024)
miR-326	↑ MS	Heart	Increases severity of MI, inducing LVSD	Serum	Abaas and Darweesh (2024)
				Whole Blood	Basak et al. (2023)
				Plasma	Al-Temaimi et al. (2024)
				*In-Vitro* T Cells	Azimi et al. (2019)
S100A1	↑ MS	Heart	Increases risk of MI	White Brain Matter	Jank et al. (2025)
SOD1	↑ ALS	Eye, Muscle	Induces visual Impairments, impaired muscle organization, and mitochondrial dysfunction	*In-Vitro* Astrocyte, Serum	Basso et al. (2013), Sproviero et al. (2018)
				*In-Vitro* Microglia	Massenzio et al. (2018)
TDP-43	↑ ALS	Muscle	Induces muscle atrophy and mitochondrial dysfunction	CSF, Serum	Feneberg et al. (2014)
				*In-Vitro* Neurons	Feiler et al. (2015)
FUS	↑ ALS	Muscle	Induces muscle atrophy and mitochondrial dysfunction	*In vitro* SH-SY5Y, N2A	Kamelgarn et al. (2016)
HTT	↑ HD	Heart, Muscle, Liver	Promotes cardiac dysfunction, impairs liver metabolism, and destabilizes muscle mitochondrial DNA	Plasma	Ananbeh et al. (2022)
				*In-Vitro* NPC	Miguez et al. (2023)

Abbreviations: EV, extracellular vesicle; MS, Multiple Sclerosis; ALS, Amyotrophic Lateral Sclerosis; HD, Huntington’s Disease; SOD1, Superoxide Dismutase 1; TDP-43, Transactive Response DNA-Binding Protein 43; FUS, Fused in Sarcoma; HTT, Huntingtin Protein; HF, heart failure; MI, myocardial dysfunction; LVSD, left ventricular systolic dysfunction; CAD, coronary artery disease; CSF, cerebrospinal fluid; SH-SY5Y, SH-SY5Y, human neuroblastoma cell line; N2A, N2A mouse neuroblastoma cell line; NPC, neural progenitor cells.

#### 3.3.1 Multiple Sclerosis

Multiple sclerosis (MS) is a chronic autoimmune disease characterized by demyelination, neuroinflammation, and neurodegeneration in the CNS. It is estimated that there are 2.8 million cases of MS worldwide, with prominent symptoms including cognitive impairment, motor issues, and fatigue in patients (Walton et al., 2020). MS pathology manifests in various clinical forms, primarily relapsing-remitting MS (RRMS) and progressive MS, and involves immune system dysfunction, particularly the role of autoreactive T and B cells, microglial activation, and oligodendrocyte damage. T cells and B cells mistakenly target the patient’s own myelin and oligodendrocytes preventing remyelination, inducing demyelination that results in lesions on the axons, eventually resulting in neurodegeneration. In addition, it has been noted that disruptions in the BBB may occur within MS due to a number of driving factors that are still being explored (Hansen et al., 2024; Nishihara et al., 2022). In RRMS, inflammatory lesions form prior to the onset of symptoms. Once symptoms develop, the patient experiences repetitive phases of aggravated neurological symptoms followed by apparent, temporary recovery. These phases may alternate for anywhere between 5-20 years until transitioning to a stage where neurological symptoms gradually deteriorate, with phases of recovery becoming more infrequent, and immunomodulatory and immunosuppressive therapies growing less effective (Hart et al., 2021). Conversely, progressive MS makes up 15% of MS cases, wherein MS pathology is progressive from the start, and gradually worsens without going through phases of relapsing and recovering (Pogoda-Wesołowska et al., 2023).

MS patients often suffer from lethal kidney and respiratory complications and infections, but are also known to have a high risk for heart failure and osteoporosis, and looser links to diabetes, largely as a result of the gradual immobilization of MS patients and the side effects of corticosteroid MS treatments (Bacchetti et al., 2025). This link to cardiovascular disease is largely believed to be a result of the demyelination of nervous tissue, but there is also evidence to suggest that BDEVs play a role. EVs during MS pathology have also been shown to carry dysregulated levels of several miRNAs that are believed to play a role in heart diseases, such as miR-29a and miR-29b (Yousuf and Qurashi, 2021), which are also known to correlate with cardiovascular diseases such as hypertrophy, atrial fibrillation, and heart failure (Lee et al., 2024; Mahjoob et al., 2022), or miR-155 (Basak et al., 2023), which is known to be released by CNS epithelial cells (Yang et al., 2022), and believed to play a role in Coronary Artery Disease (CAD) (Ahmed et al., 2021; Mahjoob et al., 2022). Many of these miRNAs, however, need further investigation to ascertain the role they play in MS, their viability as biomarkers, or their respective impacts on peripheral organs.

One of the most significant biomarkers for MS is miR-326, which is expressed at significantly higher levels in EVs in blood samples from MS patients (Abaas and Darweesh, 2024; Basak et al., 2023; Al-Temaimi et al., 2024), and is believed to play an important role in MS by determining the fate of T-cell differentiation (Basak and Majsterek, 2021). Conversely, Iglesias et al. noted that neuronal and oligodendrocyte derived EVs isolated in the blood stream showed decreased levels of miR-326 in patients who stayed in a phase of temporary recovery (Torres-Iglesias et al., 2025). Recent studies have thoroughly explored the effects of miR-326 on the cardiovascular system. Dantas-Komatsu et al. provided evidence that increases in the levels of miR-326 preceded left ventricular systolic dysfunction (LVSD) in a post-MI environment. Further investigations into the subject shows that the researchers could successfully use miR-326 to predict cases of LVSD, as well as identify individuals who were not at risk of developing LVSD using the absence of miR-326 overexpression, suggesting that miR-326 plays a role in inducing ventricular dysfunction (Dantas-Komatsu et al., 2023). He et al. similarly displays that heart transplants with high levels of miR-326 following the operation were at a significantly higher risk for acute rejection and further postoperative complications (He et al., 2023). Dogan et al. explores the direct effects of miR-326, demonstrating its impact on mitochondria biogenesis, and by extension cellular energy metabolism, highlighting the possibility that dysregulated levels of miR-326 may induce oxidative stress and exacerbate or create problematic symptoms (Dogan et al., 2024). Luo et al. similarly shows miR-326’s effect on the heart, providing evidence that exosomal miR-326 regulates FoxM1 signaling in cardiovascular muscle cells, an oncogene that plays an important role in cell proliferation and repairing DNA damage (Luo et al., 2022; Li et al., 2024a).

We would be remiss to not mention the dysregulation of proteins related to MS pathogenesis in BDEVs. Recent studies have shown that proteins involved in lipid metabolism and inflammation were upregulated, whereas a number of synaptic proteins and mitovesicle proteins were found to be downregulated in BDEVs, most of the top dysregulated proteins having been previously shown (Jank et al., 2025; Duan et al., 2024). The upregulated protein S100A1 is known for its role in regulating Ca^2+^, and has been shown to correlate with cardiovascular diseases such as MI (Fan et al., 2019). Together, these studies highlight the potential impacts that miRNA and protein enriched BDEV-mediated brain-heart crosstalk can have within an MS environment.

#### 3.3.2 Amyotrophic lateral sclerosis

Amyotrophic lateral sclerosis (ALS) is a progressive and fatal neurodegenerative disorder that primarily affects motor neurons, resulting in muscle atrophy, paralysis, and eventual death. The exact etiology of ALS remains unclear, in part due to diagnostic challenges during early stages. Symptom severity typically increases with age, with the highest incidences observed in individuals aged 60-79 (Marin et al., 2018). The number of incidences vary from region to region, but are estimated at approximately 11.8 per 100,000 in the United States (Wolfson et al., 2023). ALS is characterized by the selective degeneration of upper and lower motor neurons, causing progressive muscle weakness, spasticity, and respiratory failure, and has also been linked to cardiovascular diseases, gastrointestinal complications, osteoporosis, and metabolic diseases. Like MS, these diseases are primarily believed to be either the direct result of ALS effects on muscles in the cardiovascular and gastrointestinal systems inducing heart disease or esophageal dysfunctions, or as the result of reduced physical activity in ALS patients, increasing the risks for osteoporosis, diabetes, dyslipidemia, or other peripheral complications. Cases are classified as sporadic (sALS), which makes up 85% of cases, or familial (fALS), which makes up approximately 15% of cases (Feldman et al., 2022). ALS is commonly linked to genetic mutations in genes including TAR DNA-binding protein (TARDBP), superoxide dismutase 1 (SOD1), and fused in sarcoma (FUS), which are respectively responsible for the TDP-43, SOD1, and FUS proteins (Goutman et al., 2018). In ALS pathology, misfolded TDP-43, SOD1, and FUS proteins accumulate in motor neurons, inducing mitochondrial dysfunction, increasing oxidative stress, and blocking axonal traffic. Microglial activation and astrocyte dysfunction contribute to ALS pathology by releasing pro-inflammatory cytokines, such as Tumor Necrosis Factor α (TNF-α) and Interferon γ (IFN-γ), and increasing ROS levels, thus exacerbating neuronal damage (Guo et al., 2022; Vahsen et al., 2023).

A particular case study by Tanemoto et al. focused on a specific ALS patient with the FUS P525L mutation, revealing that the patient was suffering from asymmetric muscle weakness accompanied by anti-ganglioside antibodies, linking FUS to mitochondrial abnormalities in skeletal muscle and ultimately muscle degeneration (Tanemoto et al., 2021). These findings reinforce that skeletal muscles actively contribute to ALS progression, potentially through interactions with BDEVs carrying bioactive cargo. Although consensus on defined ALS biomarkers is lacking, several proteins, including SOD1, TDP-43, and FUS, are closely linked to the disease’s pathology. In fact, BDEV levels of SOD1, TDP-43, and FUS have all been shown to increase in ALS pathology, and are prime targets to modulate interorgan communication as BDEV-cargo (Li et al., 2023c).

SOD1 functions as an antioxidant enzyme, and plays an indispensable role in maintaining redox homeostasis. Although ALS has been classically defined as a motor neuron disease, increasing evidence suggests a broader pathological spectrum, wherein mutant SOD1 exerts toxic effects on non-neuronal cells, including the retina and skeletal muscle fibers. Augustin et al. and Merkle et al. both highlight the structural and functional deterioration in SOD1-deficient mice. These studies demonstrate that loss of SOD1 leads to increased oxidative stress, accelerated retinal aging, and degeneration of melanin-containing structures, all of which contribute to visual impairment (Augustin et al., 2020; Merkle et al., 2022). A more recent study by Verma et al. provides a comparative analysis of SOD1 mutation effects in neuronal and muscle cell populations. The findings highlight distinct vulnerabilities of muscle cells, including impaired mitochondrial bioenergetics, altered calcium homeostasis, and cytoskeletal disorganization, bolstering the argument that muscle tissue suffers from mutant SOD1 toxicity independently from CNS complications in an ALS environment, but also notes that mutant SOD1 does not build up in muscle cells when isolated (Verma et al., 2024). This suggests that SOD1 toxicity in muscle tissue is not merely a secondary consequence of motor neuron degeneration but an autonomous pathological process, and that mutant SOD1 cannot occur purely due to local musculoskeletal expression.

TDP-43 and FUS are proteins known to play roles in modulating RNA transcription and repairing DNA. While their link to ALS pathology is largely believed to be a result of their impact on the CNS, recent studies indicate that they contribute significantly to muscle degeneration. Research by Chen et al. discovered a novel mechanism by which TDP-43 directly causes mitochondrial stress in muscular tissue, demonstrating that aggregated TDP-43 influences muscle atrophy by releasing mitochondrial DNA into the cytoplasm and inducing mitochondrial dysfunction (Chen et al., 2023). Recent studies by Lynch et al. investigated TDP-43’s prion-like behavior in human and murine muscle models, finding that aggregated TDP-43 propagated misfolded conformations and exacerbated disease progression in muscle tissue (Lynch et al., 2024). Yu et al. observed that mitochondria-rich regions in muscular tissue containing mutated FUS exhibited abnormal FUS distributions and disrupted mitochondrial function, suggesting that FUS mutations have a direct effect on mitochondrial homeostasis in muscle cells (Yu et al., 2022). Alternatively, Ji et al. observed that FUS mutations inhibited myogenesis by suppressing the expression of troponin T1 (TNNT1), impairing myogenic differentiation, and leading to weakened muscle fibers, which would further exacerbate ALS-related muscle dysfunction (Ji et al., 2024).

These recent studies provide evidence that indicates that SOD1, TDP-43, and FUS mutations induce substantial pathological alterations in muscle tissue independently of neuronal degeneration. However, whether these symptoms result in part due to BDEV transported mutated or misfolded cargo, or is entirely a result of the mutated proteins being locally produced in the peripheral organ is still unclear in the case of TDP-43 and FUS. Interestingly, Gall et al. found that muscle cells from ALS patients secrete neurotoxic extracellular vesicles, contributing to motor neuron degeneration, suggesting that the ALS brain-muscle axis is bidirectional (Le Gall et al., 2022). While findings suggest that dysfunctional SOD1 potentially contributes to retinal and macular damage, it is unclear whether this is true in an ALS environment, and thus requires further investigation. Within the current field, there is a mounting pile of evidence that TDP-43 and FUS mutations have a significant impact on skeletal muscle function in ALS, however, these studies have similar issues pertaining to the potential impact of BDEV-mediated communication in ALS. It is unclear how much BDEVs contribute to the symptoms that TDP-43 and FUS supposedly cause within peripheral muscular tissue, and due to the genetic nature of these mutations, the specific contribution of BDEVs in any ALS environment remains unclear.

#### 3.3.3 Huntington’s disease

Huntington’s disease (HD) is a fatal, heritable neurodegenerative disorder characterized by the pathological expansion of the CAG trinucleotide repeat in the *Huntingtin* (*HTT*) gene, resulting in an aberrant polyglutamine (polyQ) tract within the mutant HTT protein (mHTT). The mHTT protein can aggregate and disrupt mitochondrial dynamics, impair oxidative phosphorylation, and increase ROS production, culminating in neuronal dysfunction and degeneration. The progressive nature of neuronal atrophy results in the gradual onset and worsening of motor impairments, cognitive decline, and psychiatric manifestations (Stoker et al., 2022). HD patients often suffer from lethal dysphagia and cardiac arrhythmias due to the weakening of the autonomic nervous system, and more moderate metabolic conditions and osteoporosis as a result of patients’ reduced mobility. Neuroinflammation is prevalent in HD pathogenesis, as pro-inflammatory cytokines, including TNF-α and IL-6, exacerbate neuronal injury and accelerate neurodegeneration (Gao et al., 2023a). The toxic effects of mHTT also extend beyond neurons, affecting glial cells and peripheral tissues (Saba et al., 2022).

EVs have long been implicated in facilitating the intercellular spread of mHTT; however, direct evidence differentiating brain-derived EV transport from peripheral EV populations remains limited. EVs facilitate the intercellular transfer of polyglutamine-expanded HTT protein and CAG-repeat RNA, acting as a transportation vehicle in the spread of toxic proteins, accelerating the progression of HD (Zhang et al., 2016). In HD, microglia-derived EVs have been shown to contain pro-inflammatory cytokines and other bioactive molecules that exacerbate neuronal damage and propagate inflammation, in addition to reactive astrocytes releasing EVs that influence disease progression by modulating neuronal and immune responses (Gao et al., 2023a). The ability of EVs to transport pathogenic molecules and modulate immune responses positions them as key players in HD pathogenesis. Although evidence for miRNAs as viable HD biomarkers remains limited, preliminary findings indicating mHTT presence in EVs raise the hypothesis that CNS-derived EVs could transport pathogenic cargo to peripheral tissues. However, there is a need for direct experimental validation confirming BDEV-specific transport.

Recent studies found that mHTT originating from the CNS are capable of crossing the BBB and travel through the bloodstream. Further exploration proved that CNS-derived mHTT can aggregate within peripheral organs including the heart, liver, pancreas, and kidney (Chuang and Demontis, 2021; Rieux et al., 2021; Gómez-Jaramillo et al., 2022). However, these studies have not conclusively demonstrated direct mechanistic transfer by BDEVs. Circulatory system connection experiments showed observable mHTT aggregates in the wild type mouse, and reduced levels of mHTT in the diseased mouse, accompanied by increased and decreased HD symptoms respectively (Rieux et al., 2021). These experiments do not differentiate between brain and peripheral derived mHTT, or rule out alternative systemic transfer mechanisms, leaving questions concerning the role of EVs.

It is well recorded that individuals with HD have a significantly higher risk of cardiovascular diseases, even when compared to unaffected first-degree relatives, suggesting that HD pathology, and potentially mHTT, contribute to cardiac dysfunction (Sørensen and Fenger, 1992). Additionally, silencing the HTT gene, and reducing mHTT expression alleviated cardiac dysfunction, supporting the theory that mHTT exerts direct cardiotoxic effects (Park et al., 2021). Studies in the BACHD mouse model, performed by Schroeder et al., showed that HD mice exhibited significant cardiac dysfunction very early on, prior to the progression of neurodegeneration and neuronal dysfunction, further supporting the notion that mHTT induces cardiac dysfunction through an alternative pathway (Schroeder et al., 2016). Conversely, early-stage dysregulation of the autonomic nervous system observed in HD complicates interpretations of cardiac dysfunction, highlighting the difficulty in distinguishing direct effects of mHTT transported by EVs from broader systemic neurodegeneration (Kiriazis et al., 2012; Schroeder et al., 2016). This underexplored avenue may contribute to the increased incidences of cardiovascular disease in HD patients, and in turn makes it increasingly difficult to establish the direct contribution of BDEV-transported mHTT.

Past studies have shown that, in addition to significant levels of protein aggregates accumulating in the liver in HD, mHTT disrupts transcriptional regulation in hepatocytes, targeting genes involved in lipid metabolism, gluconeogenesis, and aggregate clearance. Investigating HD R6/2 mouse models provided evidence that these deficits are a result of mHTT’s interaction with multiple transcription factors, reducing PEPCK activity and impairing PGC-1α signaling (Josefsen et al., 2010). More recent studies from Bragg et al. suggest that the loss of wild-type HTT in hepatocytes results in multiple physiological changes, including increased bile acids, cholesterol, hypoglycemia, impaired cell adhesion, and a clear shift in liver zonation, elucidating the roles HTT plays within the liver (Bragg et al., 2023). It is well known that muscle tissues in HD models exhibit similar symptoms to neurons, that is fragmented mitochondrial function and the associated deficits that occur with it. Recent studies performed by Neueder et al. on muscle biopsies from HD models reveal that mHTT directly affects mitochondrial DNA in muscle tissues (Neueder et al., 2024). Chivet et al. similarly found that the lack of functional HTT resulted in large calcium fluctuations as a result of HTT’s interactions with junctophilin 1 and stromal interaction molecule 1 (Chivet et al., 2023). These studies primarily discussed the known peripheral impacts of mHTT; however, explicit investigations distinguishing BDEV-mediated cargo transport from other systemic EV populations remain insufficient, showing there is a critical translational gap within Huntington’s Disease environment.

## 4 Perspectives and future directions

This review article examines the mechanics and effects that BDEV-mediated interorgan communication has on peripheral organs in neurodegenerative diseases. Numerous studies demonstrate that extracellular vesicles play an important role in interorgan communications, and that extracellular vesicles derived from the brain regularly communicate outside of the nervous system, having an unidentified impact on the body’s biological systems. EVs, in addition to making for promising prospective diagnostic and prognostic biomarkers, are a potential mechanism by which diseases such as Alzheimer’s Disease, Parkinson’s Disease, and other NDs affect the heart, kidney, and other organs implicated with these diseases. During the pathology of these NDs, EVs have been shown to carry significant amounts of hallmark proteins, such as Aβ_42_ and α-syn, as well as dysregulated levels of various non-coding RNAs, each of which is known to have their own protective or detrimental effects within different organs. The EV-mediated pathogenic transportation of biologically potent cargo from the brain to peripheral organs may explain a facet of the systemic nature of neurodegenerative diseases, which are known to impact and cause complications outside of the nervous system in patients.

As discussed earlier, in an AD and PD pathological environment, BDEVs have been shown to regulate the differentiation of BMSCs by carrying signaling miRNAs that modulate adipogenesis and osteogenesis related pathways. Recent studies reported that targeting EV contents, can either promote or inhibit adipocyte formation (Zhong et al., 2021). Within the current field, EVs have frequently been investigated for their therapeutic potential, with studies utilizing EVs to improve cardiac function and restore cardiac repair mechanisms in a diseased environment. Similar studies performed in a PD cardiovascular environment utilized EVs engineered with cardioprotective properties to counteract mitochondrial dysfunction and mitigate cardiac hypertrophy (Lu et al., 2023). Further research identified additional cardioprotective mechanisms associated with these EVs, including their ability to reduce oxidative stress and modulate immune responses in cardiac tissues. These therapies can reverse the harmful effects that we’ve observed to correlate with the dysregulated cargo that BDEVs transport within an ND environment, or in some cases, outright preventing common complications from arising in patients. Their findings underscore the therapeutic potential of EVs in NDs, and further elucidating the mechanisms behind EV-mediated osteoporosis and cardiovascular diseases may pave the path for developing novel treatments in ND patients.

In Parkinson’s Disease, miR-124 has recently received more attention as a biomarker with therapeutic potential. As previously discussed, miR-124 is a microRNA that is downregulated in PD BDEVs which normally serves in a cardioprotective capacity but loses its protective effects and can exacerbate cardiovascular diseases in PD pathology. Studies have highlighted the dual function of miR-124 as both a biomarker and a neuroprotective agent, as miR-124 modulates the expression of inflammatory cytokines such as TNF-α and IL-6 in addition to its reported cardioprotective effects (Ghafouri-Fard et al., 2021). BDEVs enriched with miR-124 have demonstrated the ability to enhance neuroprotection in PD mice, modulating neuroinflammation and promoting neuronal repair, and can be engineered to enhance tissue-specific targeting (Esteves et al., 2022). Recently, EVs enriched with miR-124-3p have been used to treat IS injuries, mediating oxidative stress, and protecting cardiomyocytes, counteracting many of the negative symptoms previously reported to be a result of the dysregulation of miR-124 in PD BDEVs. The development of miR-124-enriched EVs as a treatment option represents a cutting-edge approach for treating NDs. With continued advancements in identifying therapeutic targets and elucidating their mechanisms, EV-based therapies could become a cornerstone of regenerative therapeutics, offering new hope for patients with neurodegenerative diseases.

Despite their promise, there are multiple challenges that complicate the analysis of BDEVs, the exploration of clinical applications and therapies utilizing BDEVs, and the treatments for BDEV dysregulations. A limitation in the current field is the lack of direct evidence confirming the CNS origin of many EVs detected in peripheral fluids like blood plasma. As a result, while some conclusions may suggest contributions from brain derivatives, definitive proof of BDEV-mediated cargo transfers is still limited and needs further validation. Continued investigations into optimizing BDEV-based diagnostics, reducing variability in EV composition and EV isolation methods, are necessary to enhance the reproducibility of results.

Additionally, different isolation protocols for EVs can yield distinct EV populations, which can complicate comparisons between studies. Currently, studies use different enzymes (papain vs. collagenase), mechanical vs. enzymatic disruption, filtration timing, and purification strategies that can vary the type, purity, and size of the EVs, potentially even disrupting contaminant levels. Particularly, some protocols (especially tissue dissociation) may unintentionally generate non-EV structures which could contaminate final EV-prep and skew proteomic results (Brenna et al., 2021). Differences in isolation methods may introduce inconsistencies in EV purity, size, and composition that hampers direct comparisons made across studies. Therefore, standardization or method-specific benchmarking is likely necessary to make reliable comparison and currently poses major translational challenges.

Furthermore, future investigations should focus on providing direct evidence supporting the impact of BDEV-mediated communication between the brain and other organs, as well as alterations in BDEV-induced behavior in an ND environment. The precise mechanisms by which BDEVs mediate interorgan communication, and their contributions to disease progression outside of the CNS, are not yet completely understood. Future studies must also continue to investigate the specific cargo of EVs in both healthy and diseased states to identify reliable biomarkers and potential therapeutic targets. The contributions of pathological proteins, such as Aβ_42_ and α-syn, to complications outside of the CNS in the disease state of NDs need further investigation so that effective treatments can be formulated. Systemic complications in NDs often extend beyond direct inflammatory effects in the brain and can influence not only the production and uptake of circulating EVs, but also the cargo of BDEVs. For example, proinflammatory cytokines which are elevated in metabolic dysfunction, have been shown to stimulate EV release from neurons and glial cells and to modulate their miRNA and protein (Al-Mansoori et al., 2022; Pistono et al., 2020). Additionally, insulin resistance and oxidative stress, characteristics of metabolic dysfunction, can alter neuron EV composition and increase the EV-associated inflammatory cargo (Freeman et al., 2018). Future studies should explore how these changes may influence the way BDEVs interact with peripheral tissues, potentially increasing their uptake in organs affected by metabolic stresses and contributing to systemic manifestations of NDs. The miRNAs and proteins reviewed in this article provide a promising list of targets and potential players that influence peripheral organs in the disease state of AD, PD, and other NDs; however, their effects need to be further explored and confirmed to develop a more comprehensive understanding.
